# Novel Immune Stimulant Amplifies Direct Tumoricidal Effect of Cancer Ablation Therapies and Their Systemic Antitumor Immune Efficacy

**DOI:** 10.3390/cells10030492

**Published:** 2021-02-25

**Authors:** Mladen Korbelik, Tomas Hode, Samuel S. K. Lam, Wei R. Chen

**Affiliations:** 1 Integrative Oncology Department, BC Cancer, Vancouver, BC V5Z 1L3, Canada; 2 Immunophotonics Inc., St. Louis, MO 63110-1110, USA; tomas@immunophotonics.com (T.H.); samuel@immunophotonics.com (S.S.K.L.); 3 Stephenson School of Biomedical Engineering, Gallogly College of Engineering, University of Oklahoma, Norman, OK 73019, USA

**Keywords:** N-dihydrogalactochitosan (GC), IP-001, tumor ablation, immunoadjuvant, immune stimulant, metastatic tumors, combination of ablation and immune stimulation, interventional immuno-oncology (IIO)

## Abstract

Ablation therapies have emerged as an effective tool for destroying cancerous tissue, but for advanced and disseminated tumors their application remains mainly a palliative measure. However, it is becoming increasingly clear that this limitation can be redressed by the use of intratumoral immune stimulating agents for amplifying potential antitumor immune responses that are induced by ablation therapies. A novel immune stimulating drug IP-001, a specific variant of the N-dihydrogalactochitosan (GC) family of molecules, has shown to be effective against metastatic tumors, when combined with different forms tumor ablation. It acts as a multi-function immune stimulant both by directly inhibiting cell membrane repair and recycling of ablation-damaged tumor cells, and indirectly by sequestering ablation-released tumor antigens, as well as recruiting and stimulating antigen presenting cells to induce a potent Th1 type T cell response against the cancer. In this review, we briefly discuss the current applications of local ablation for cancer treatment and the effects of GC in combination with other ablation therapies, a therapeutic approach that is pioneering the field of Interventional Immuno-Oncology (IIO).

## 1. Introduction

A significant expansion of immunotherapeutic approaches established in recent years has conclusively contributed to improved survival rates for several types of cancer [1]. To continue this trend, novel strategies for harnessing potent and durable antitumor immune responses are contemplated. One such example is presented in the present paper.

## 2. Tumor Ablation Therapies and Antitumor Immunity

Various tumor ablation therapies are playing increasingly important roles in interventional oncology. They are performed by a direct local application of energy and/or chemical to the targeted tumor aiming for its rapid in situ destruction. Technologies used for this purpose include various forms of thermal energy delivery, non-thermal illumination, radiation, and electric field exposure [2,3,4]. Positive characteristics of tumor ablation therapies include their minimally invasive nature, absence of serious systemic side-effects, and good healing of surrounding normal tissue. 

An important benefit common to the tumor ablation therapies is breaking the immunotolerance of the host to the treated tumor orchestrated by local thermal, oxidative or electric stress [5,6]. Temperature stress (hot or cold) is induced by treatments like microwave ablation, radiofrequency ablation, photothermal therapy (PTT), cryoablation therapy (CAT), and high frequency ultrasound [3]. Oxidative stress is induced by photodynamic therapy (PDT) [7], and electric stress by irreversible electroporation (IRE) and other forms of electric based ablation [3]. In all cases the inflicted rapid insult is perceived as a local acute trauma at the targeted lesion. The predominant damage is misfolded dysfunctional proteins accumulated in the cytoplasm of tumor cells at the treated site that threaten tissue homeostasis, particularly the protein homeostasis called proteostasis [8]. This compels the host to launch a canonical protective response for maintaining the integrity of proteome known as the proteostasis networks that operates by integrated stress signaling cascades [9]. The involved signal transduction systems encompass four groups of pathways:

i. Rectifying changes in gene and protein expression at transcriptional and translational levels (best known are heat shock response, integrated stress response, unfolded protein response, and antioxidant response);
ii. Cellular membrane repair responses engaging sterol regulatory element-binding proteins (SREBPs) pathway mediating the control of cholesterol and fatty acid metabolism and caspase-1 activity for maintaining cellular integrity (to be discussed below);
iii. Improving disposal of damaged proteins and cells (including ERAD (ER-associated protein degradation response), autophagy, and various cell death signaling responses);
iv. Inflammatory-immune and other cell non-autonomous responses (including DAMPs signaling, NF-κB activation signaling, Toll-like receptor (TLR) upregulation signaling, heat shock protein signaling, immunogenic cell death signaling, immunoregulatory cell signaling) [10].

Another critically important element is that stress response triggered in cells of tumors treated by tumor ablation therapies increases their immunogenicity as they turn up expressing cryptic tumor antigens due to their changed (unconventional) translational activity [10,11]. On the other hand, compromised integrity of the cell membrane and final disintegration of the cells (either through coagulative necrosis or other cell death mechanisms) caused by the aforementioned physical energy contribute to release of tumor-associated antigens.

## 3. The Family of N-Dihydrogalactochitosans as Promising Immune Stimulating Drugs

It has become increasingly evident that a robust and prolonged antitumor response (and hence effective overall clinical outcome) can be obtained by tumor ablation therapies when combining them with appropriate adjunct agents promoting tumor antigen delivery and enhancing antitumor immune response. Our work has shown that one of the most promising agents for such combination is semisynthetic cationic carbohydrate biopolymer N-dihydrogalactochistosan (GC), particularly IP-001, a well-characterized variant of GC. Synthesized and purified under GMP conditions, IP-001 characterization includes the testing for endotoxins, metals, and other impurities which eliminates a major confounding factor in the research of naturally derived molecules. Water solubility, sterile filterability, biocompatibility, non-toxicity, and capability to serve as physiologically compatible carrier (e.g., nanomaterial vehicle) are key advantageous properties of IP-001 [12,13].

Originally, GC was prepared as functionalized chitosan by attaching galactose molecules to this linear polysaccharide, which itself is a derivative of an abundant natural polysaccharide chitin [12,14].

Previously reported immunostimulatory properties of GC include promoting immune activity of macrophages and dendritic cells (DCs); upon phagocytizing GC these cells enhance their uptake of tumor antigens and boost their antigen-presenting effectiveness [13,15].

## 4. GC Combined with Thermal Ablation and Photodynamic Therapy

We have investigated the effects of combining GC with four types of tumor ablation therapy: laser-based photothermal therapy (PTT), CAT, high-intensity focused ultrasound (HIFU) and PDT in various pre-clinical studies [14,16,17]. In all cases GC was found to enhance antitumor efficacy of these therapies. The PTT treatment used near-infrared lasers (around 800 nm) to deliver thermal energy focused on targeted rat or mouse tumors by means of optical fiber either topically or via interstitial active lens tip; [14,16]. For PTT in vitro, tumor cell pellet was exposed superficially to infra-red diode laser light from the tip of a 100 µm multimode optical fiber [18]. In our PDT studies, tumors on mice injected with photosensitizer were illuminated superficially with red light generated by a QTH lamp and delivered by a liquid light guide [14,16]. For PDT treatment in vitro, cells previously incubated with a photosensitizer (temoporfin, Photofrin, or chlorin e6) were exposed to the light from the same source filtered for the wavelength matching the photosensitizer absorption peak [16]. For both studies we employed also therapeutic tumor vaccine protocols, where mouse SCCVII tumor cells treated by PTT or PDT in vitro were injected as a vaccine material peritumorally into mice bearing established SCCVII tumors [16,18]. For CTA treatment, we tested the in vitro model where tumor cells were exposed to −80 °C temperature for pre-set time intervals [16]. The GC treatment was most frequently applied immediately after PDT/PTT light or CAT −80 °C exposure.

Numerous other studies have demonstrated safety and efficacy of GC in different cancer models [13,19,20,21,22,23]. For example, in a recent study using a pancreatic cancer model [20], mice were injected with Panc02-H7 tumor in both flanks, but only one tumor was treated with thermal ablation plus IP-001 injection. Contralateral tumor harvested at 7 days showed significant increase in CD3^+^CD8^+^IFNγ^+^ and CD3^+^CD4^+^IFNγ^+^ T cells over ablation alone and untreated control, while T-regulatory cell population decreased in frequency. Peripheral blood and supernatant from splenocytes re-stimulated in vitro both showed significantly increased levels of IFNγ and IL-12 (in the serum) when IP-001 was added to ablation compared to all monotherapy controls. The increase of tumor-infiltrating and IFNγ-secreting CD4^+^ and CD8^+^ T cells, and the elevation of key Th-1 and CTL (cytotoxic T lymphocyte)-driving cytokines IFNγ and IL-12, together reveals that IP-001 favors the initiation of cytotoxic T lymphocytes with T-helper type 1 response. Furthermore, the data indicates that the effect is of significant magnitude to eliminate both primary tumors and metastatic disease in solid cancer models. This type of T cell immunity has been established to be one of the most potent antitumor immune response described in the literature [24]. Ultimately, this has important implications since strategies that amplify CTL responses theoretically also benefit from the re-invigorating effects of checkpoint blockade therapies.

Our studies have uncovered additional mechanisms of immunostimulatory effects of GC exerted by hindering the rise in immunoregulatory cell levels associated with response to tumor ablation therapies mediated through stress signaling networks involving master transcription factor signal transducer and activator of transcription 3 (STAT3) [25,26]. The rise in levels of both major populations of these cells, myeloid-derived suppressor cells (MDSCs) and regulatory T cells (Tregs), detected after tumor treatment by either PDT or PTT was greatly reduced when these therapies were combined with GC treatment [16,18]. Hence, GC can promote immune activity both directly by promoting the activity of cells like macrophages and DCs, and indirectly by reducing the numbers of immunoregulatory cells. This latter property is particularly important because the quality, magnitude, and persistence of immune response (including antitumor immune response) is governed by the net balance of promoting and dampening immune response, the latter by built-in restricting influences evolutionary developed as mechanisms for maintaining immune homeostasis. Curing the tumor requires blocking this natural balancing action, and agents like GC could be exploited for such a task.

To put in perspective the benefit of GC in combination with photothermal therapy, the therapeutic impacts of GC were compared with several benchmark immunoadjuvants in the treatment of DMBA-4 mammary tumors in rats [14]. DMBA-4 tumors are poorly immunogenic and highly metastatic. Three commonly used immunoadjuvants, complete Freund’s (CF) adjuvant, incomplete Freund’s (IF) adjuvant, and Corynebacterium parvum (CP), were mixed with indocyanine green (ICG) and intratumorally injected into DMBA-d mammary tumors in rats, followed by non-invasive irradiation of an 808-nm laser light [14]. The absorption peak of ICG (around 800 nm) matched the laser wavelength, leading to effective thermal destruction of local tumors. The immunoadjuvants provided limited antitumor effect with the long-term survival rates of 19%, 7%, and 9%, for CF, IF, and CP, respectively. However, in comparison, laser-GC induced anti-tumor immune responses were stronger than that of the three immunoadjuvants, leading to a 29% long-term survival [14].

## 5. Potentiation by GC of Direct Tumoricidal Effect of Tumor Ablation Therapies

Perhaps the most extraordinary effect of GC discovered in our recent studies is its capacity to directly affect the viability of tumor cells that sustained the damage by tumor ablation therapies. This was demonstrated by determining the survival of tumor cells treated in vitro by PTT, PDT or CAT immediately plated with or without presence of GC for cell colony formation assay [16,18]. The results consistently showed that GC markedly increased the death rates of tumor cells treated with various forms of ablation therapy. For this effect, the presence of GC was critical for the initial hours after PDT or equivalent treatment and a direct positive correlation was found with GC dose. With PDT, as with CAT, this effect was seen with different therapy doses as well as with three different photosensitizers [16].

Since exposure to GC selectively killed ablation therapy-treated cells and not cells exposed to GC alone, the possibility of a difference in the extent of GC binding was examined by fluorescence microscopy. For this purpose we used GC conjugated to FITC as described earlier [13]. Mouse SCCVII tumor cells treated with PDT or PTT were immediately transferred into medium containing GC-FITC. The determination of FITC-based fluorescence on cells collected 3 h later revealed a significant preference of GC for binding to PDT- or PTT-treated cells compared to untreated control cells [16,18]. Since no significant cell fluorescence was found with cells exposed to free FITC, it is clear that the cell binding was mediated by GC. The fluorescence patterns in obtained cell images were consistent with the localization of GC-FITC on cellular surface. Importantly, binding of GC on PTT- or PDT-treated cells was blocked by prior exposure of these cells to annexin V [16,18]. This reveals that annexin V can compete with GC for the same interaction sites on the cell surface. Annexin V has a high predisposition for binding to acidic phospholipids with especially strong affinity for phosphatidylserine [27]. Positively-charged GC should also have similarly strong affinity to phosphatidylserine and other negatively charged membrane phospholipids that become exposed on the surface of cells (potentially early apoptotic) that sustained damage from ablation therapy treatment. The binding of large GC molecule could interfere with the repair of damaged membrane structures. This is supported by the finding that pre-exposing PDT-treated cells to annexin V before adding GC completely blocked the effect of GC on survival of these cells [16].

This capacity for preferential binding to ablation therapy-treated cells GC shares with other potent pro-immune agents like calreticulin and heat shock protein 70 [28,29]. The avidity for binding to tumor cells sustaining insult/injury from such therapies would be instrumental for the delivery of GC and other pro-immune agents to cancerous lesions for performing effective antitumor activity and their prolonged retention at tumor site. Such binding could serve as a trigger for the preferential engulfment of involved tumor cells by phagocytes with consequent tumor antigen processing and presentation for the adaptive immune response.

Further investigation of this direct cell killing mechanism of GC was directed at a possible link with cell death pathways. The possibility of bound GC acting as a ligand for one of transmembrane death receptors was not supported, because the presence of the peptide antagonist of FAS/FasL Kp7-6 or small peptide Z-IETD-fmk that selectively inhibits cspase-8 (key caspase in this pathway) had no significant impact on the survival of PTT and PTT + GC treated cells [18]. 

Next examined was a possible involvement of caspase-1 and pyroptosis in the effect of GC. Pyroptosis is a cell suicide lytic mechanism engaging members of gasdermine gene family that upon activation (primarily involving caspase-1) form oligomeric death-inducing pores in plasma membrane [30]. One of known inducers of pyroptosis is heat stress [31]. However, the experiments with both PTT- and PDT-treated cells revealed that caspase-1 has a pro-survival role because the survival of PTT+GC and PDT+GC cells decreased in the presence of specific caspase-1 inhibitor INF4E [16,18]. Several mechanisms are known to be mediated by caspase-1 to support cell survival. They include activating transcription factors such as NF-κB or influencing lipid metabolism pathways to facilitate damage membrane repair [32]. Although the exact details remain to be determined, one hypothesis is that the GC bound to membranes of ablation treated tumor cells may promote their death by hindering the repair of damaged membrane structures promoted by caspase-1 activity linked to the stress signaling-mediated upregulation of SREBPs (central regulators of cellular membrane biogenesis) [33,34]. In support of this mechanism, we found that specific inhibition of SREBPs by fatostatin A greatly reduces the survival of PDT+GC treated cells similar to INF4E [16].

## 6. Clinical Application of GC

In an investigator-initiated Phase 1 clinical trial in Peru, 10 ethnic Peruvian breast cancer patients who had failed or refused other available modalities were enrolled in Hospital Nacional Edgardo Rebagliati Martins in Lima, Peru. ECOG performance status of all patients was less than or equal to 1 at enrollment [35]. The clinical trial used localized thermal laser tumor ablation, immediately followed by intratumoral injection of IP-001. The selected mode of local tumor ablation was topical laser application combined with a light-absorbing dye (0.25% indocyanine green solution). This method of ablation was intended for a minimally invasive treatment to selectively ablate targeted tumors. However, it was found that topical burns could be significant, which suggested that interstitial photothermal applications may be better suited for subcutaneous and deep-seated lesions.

Each patient received at least one ablation + IP-001 treatment. Two patients withdrew from this study for unrelated reasons. The median age of the breast cancer patients was 52.5 years (36 to 85 years). Five patients had AJCC stage III and five patients had stage IV diseases. Three patients were diagnosed with triple-negative breast cancer. Three patients had prior surgery. Seven patients had received prior systemic chemotherapy, six patients had received radiation therapy, and five patients received hormonal therapy. Three patients did not receive any prior treatment.

The dose of GC per treatment occasion depended on tumor size, with 5 mL of 1% N-dihydrogalctochitosan being the maximum dose in a single treatment occasion [35]. The type of adverse effects (AEs) observed at the treatment site consisted of redness, blistering, stinging, burning, swelling, pain, and the formation of a hard lump or nodule at the injection site, all of which were attributed to the ablation procedure by the treating physicians. Pain and swelling reactions were noticeably stronger in patients that had received radiation therapy in the same area as the ablation plus IP-001 treatment, but in most cases, the effect had subsided significantly within 48 h. The AEs reported by treating physicians were otherwise consistent with nonclinical toxicology studies of thermal ablation combined with GC and were mild to moderate in severity. No drug-related serious adverse events were reported.

Of the 8 breast cancer patients available for evaluation, 1 patient achieved a complete remission (CR), 4 patients achieved a partial remission (PR) and 1 achieved stable disease (SD), as shown in Table 1. All local lesions responded to ablation + IP-001. In addition, metastases fully regressed in one patient and partially regressed or became stable in another five patients. 

**Figure 1:**
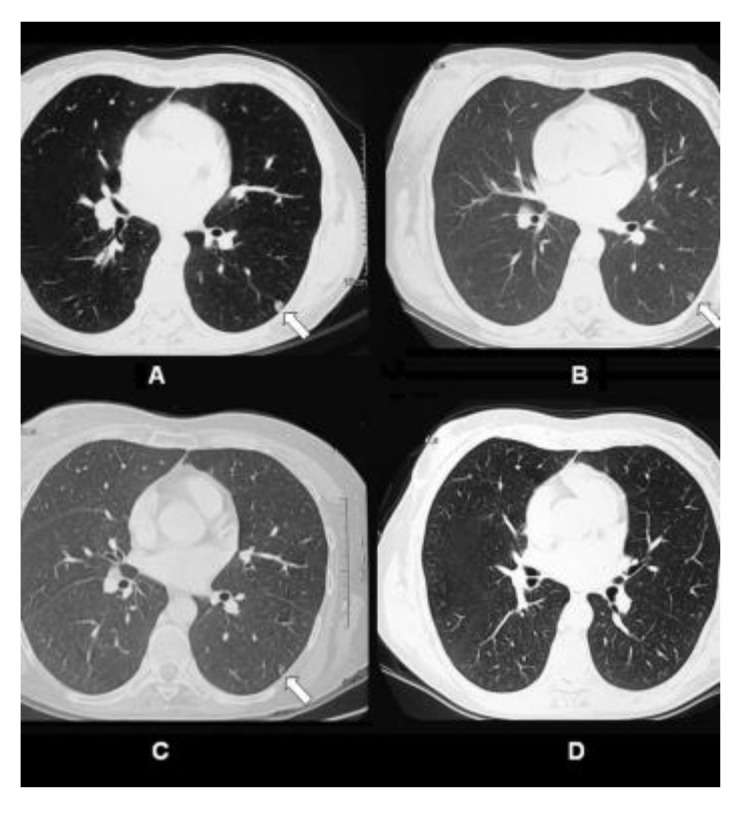
CT scans of pulmonary metastatic nodule in the left lung of 47-year-old female patient with stage IV breast cancer treated by laser ablation and IP-001 application. (**A**) CT scans of the patient taken before the first treatment. Small metastatic nodule was located in the left lung of the patient (indicated in arrow). (**B**) CT scans taken 1 week after the first treatment. (**C**) CT scans taken 2.5 months after the first treatment. (**D**) CT scans taken 12 months after the first treatment. This figure is reproduced from [35], with permission by the Royal Society of Chemistry and Owner Societies.

Case Highlight: In the one patient who achieved complete response all her pulmonary metastases disappeared. The patient was a 47-year old female diagnosed with stage IV breast cancer. She had received prior chemotherapy with AC (doxorubicin/cyclophosphamide) for 4 cycles, paclitaxel for 3 cycles, capecitabine plus ixabepilone for 3 cycles, and hormonal therapy with tamoxifen. However, she was resistant to these therapies. The patient received 4 treatments of ablation + IP-001 in total. Clinical observations before ablation + IP-001 showed that the sizes of the two tumors in the right breast of the patient were 6 × 4.5 cm and 2 × 2 cm. Metastatic nodules were observed in both lungs. Figure 1 shows the gradual regression, and subsequent elimination, of one of the patient’s lung metastases located in the left lung (indicated by arrow), before, during, and after ablation + IP-001. 

The anecdotal results from this investigator-initiated trial in patients with advanced and metastatic breast cancer, suggest a possible abscopal effect. Considering these were no-option patients, who have failed all the available treatment modalities, the effect of IP-001 was promising. Furthermore, the combination of local tumor ablation and application of IP-001 had an apparent impact on untreated, distant metastases (abscopal effect). Shown in Figure 1 are the images of a patient’s lungs after the treatment. Within a year after the treatment of breast tumors, the lung metastases completely disappeared.

## 7. Conclusions

Combining the local tumor ablation with active stimulation using GC ensures the development of immunogenic cell death with abundance of tumor-specific antigens available for immune recognition. By its nature, this represents in situ tumor vaccination [21,23].

GC enhances clinical efficacy of rapid ablation tumor therapies by both enhancing their direct malignant cell tumoricidal potency as well as amplifying the potency and durability of the induced antitumor immune response.

Clinical potential of the combination joining GC with tumor ablation therapies is supported by promising results both via pre-clinical studies and preliminary investigator-initiated Phase I safety data followed by ongoing clinical work. 

## Figures and Tables

**Table 1:** Clinical characteristics of breast cancer patients treated with ablation + IP-001 in Peru.

Patient #	Age	AJCC Stage	ER	PR	HER2/Neu	Surgery	Chemo	Radiation Therapy	Hormonal Therapy	Best Overall Response
1	71	III	+	+	+	No	Yes	Yes	Yes	NE
2	47	IV	+	+	−	No	Yes	No	Yes	CR
3	43	III	−	−	−	No	Yes	Yes	No	PD
4	36	III	+	+	-	No	Yes	Yes	No	NE
5	40	IV	−	+	+	Yes	Yes	Yes	Yes	PR
6	85	IV	−	−	−	No	No	No	No	PR
7	78	III	Unk	Unk	Unk	No	No	No	No	PR
8	58	III	−	−	−	No	No	No	No	PD
9	66	IV	+	+	Unk	Yes	Yes	Yes	Yes	PR
10	39	IV	−	+	−	Yes	Yes	Yes	Yes	SD
